# A Wavelet Packet Transform and Convolutional Neural Network Method Based Ultrasonic Detection Signals Recognition of Concrete

**DOI:** 10.3390/s22103863

**Published:** 2022-05-19

**Authors:** Jinhui Zhao, Tianyu Hu, Qichun Zhang

**Affiliations:** 1 Zhejiang-Belarus Joint Laboratory of Intelligent Equipment and System for Water Conservancy and Hydropower Safety Monitoring, College of Electrical Engineering, Zhejiang University of Water Resources and Electric Power, Hangzhou 310018, China; s1801081103@cjlu.edu.cn; 2 College of Mechanical and Electrical Engineering, China Jiliang University, Hangzhou 310018, China; 3 Department of Computer Science, University of Bradford, Bradford BD7 1DP, UK; q.zhang17@bradford.ac.uk

**Keywords:** concrete, ultrasonic detection, convolutional neural network, intelligent recognition

## Abstract

This paper proposes a new intelligent recognition method for concrete ultrasonic detection based on wavelet packet transform and a convolutional neural network (CNN). To validate the proposed data-based method, a case study is presented where the K-fold cross-validation was adopted to produce the performance analysis and classification experiments. Moreover, three evaluation indicators, precision, recall, and F-score, are calculated for analyzing the classification performance of the trained models. As a result, the obtained four-classifying CNN reaches more than 99% detection accuracy while the lowest recognition accuracy is not less than 92.5% on the testing dataset for the six-classifying CNN model. Compared with the existing stochastic configuration network (SCN) models, the presented method achieves the design objective with better recognition performance. The calculation results of the six-classifying and five-classifying models and related research clearly indicate the remaining challenging tasks for intelligent recognition algorithms in extracting features and classifying mass data from various concrete defects precisely and efficiently.

## 1. Introduction

It is known that concrete is a non-uniform building material made of cement, sand, water, coarse-grained materials and so on [1]. Image-based concrete surface defect detection can realize automatic positioning [2,3], but internal defects are still difficult to detect. Currently, ultrasonic technology is widely used in the detection of internal defects in concrete because of its strong penetrability and high sensitivity [4,5]. Ultrasonic detectors applied in the actual project judge health statuses of test objects by analyzing ultrasonic information, the propagation time, amplitudes, etc. of the ultrasonic pulse velocity [6]. This method is primarily based on physical information from ultrasonic pulses and has high accuracy when detecting homogeneous media such as metals [7]. However, the heterogeneity of concrete causes more noise or serious waveform distortion in ultrasonic detection signals. This creates difficulty in obtaining accurate detection results within the recognition results, using recognition methods based on ultrasonic echo waveforms. Moreover, recognition of signal characteristics requires good technology and experience on the part of the inspectors, and has low detection efficiency and accuracy [8]. 

More recently, there have been numerous applications of machine learning intelligence algorithms to identify ultrasonic detection signals based on signal processing methods in the time and frequency domains, combining efficient feature extraction methods. Oh [9] used a support vector machine to identify the strength grades of concrete based on ultrasonic detection signals, but the classification accuracy was less than 90%. Saechai [10] used a novel method based on a support vector machine to identify defective concrete ultrasonic detection signals, and its classification accuracy was higher than 93%. Furthermore, the experimental concrete blocks used in these studies did not add coarse aggregates and the artificial defect sizes were large, causing detection echo signals to have a relatively considerable difference based on practical concrete engineering. Hu [11] used a genetic algorithm optimized back propagation neural network to identify the ultrasonic signals of concrete defects and the average recognition accuracy is 91%. Zhao [12] used the stochastic configuration network to identify the ultrasonic detection signals of concrete defects and the accuracy is around 95%. Their experimental data were collected from concrete blocks of C30 class containing hole defects using the ultrasonic testing system [13]. These machine learning methods do not easily devise higher recognition accuracy for complex ultrasonic detection signals of either small or medium-sized defects in concrete [14].

Currently, deep learning algorithms are the most popular pattern recognition methods to perform specific tasks in more and more fields. Deep learning has the advantages of strong learning ability, good generalization, and convenient transplantation, but also has the disadvantages of large data demand and long training time [15]. As computer performance increases, these will no longer be major challenges. However, in the field of ultrasonic inspection, the application of deep learning algorithms is focused on image recognition after ultrasonic imaging, acquiring complex imaging algorithms to pre-process the detection signals [16]. The one-dimensional convolutional neural network (1DCNN) algorithms, can be directly used for signal classification. For instance, 1DCNN algorithms were applied to the fault diagnosis of bearing vibration signals [7] and the abnormality discrimination of heart sound signals [17], and their recognition accuracies were up to more than 99%. Simultaneously, Munir [18] applied a CNN to the classification of ultrasonic detection signals of metal welds and proved that the method is effective for defect recognition of ultrasonic signals. Compared with homogeneous materials, the ultrasonic detection signals of concrete contain more mixed and disturbing noise signals [19], such as reflected waves and transverse waves. In general, detection signals can be denoised before CNN performing classification and recognition computation. Fourier transform (FT), empirical mode decomposition (EMD) and wavelet package transform (WPT) [20,21,22] are commonly used frequency-domain methods for ultrasonic signal processing. Comparatively, wavelet transform is one of the most widely used methods in ultrasonic detection signal processing of concrete because of its improved time-frequency analysis ability [23,24]. 

For the purpose of developing an intelligent analysis and automated recognition of concrete ultrasonic defect signals, an effective method for WPT and CNN is proposed to diagnose the ultrasonic fault signal, whereafter the received ultrasonic detection signals are processed by a specific WPT sub-algorithm, and a specifically trained CNN model is used to automatically identify the types of concrete defects. The proposed method comprises WPT sub-algorithms to reconstruct fundamental frequency signals from ultrasonic detection signals, which provides coefficients in the first node of the third layer after three-layer decomposition of the wavelet packet. A specific 1DCNN sub-algorithm of eight layers altogether performs extracting features from the processed A-Scan signals, reducing the effective features loss or inaccurate feature extraction caused by artificial feature extraction, and realizing the automatic classification of concrete defect types. In addition, we operated the stochastic configuration network method (SCN) [24] for comparison and performance evaluation. On the other hand, the K-fold cross-validation method is applied to the classification experiment to verify the contingency of a single experiment result and reflect the performance of the model more accurately; even CNN algorithms possess the computational structure to prevent overfitting and underfitting. Through the ultrasonic detection signal classification experiment, three types of hole defects and no defect of a C30 class concrete block and a concrete block containing crack and foreign body defects are detected. Then, these detected experimental datasets are imported into the proposed method to verify their effectiveness, accuracy rate, etc. Simultaneously, the indicators are obtained to evaluate the algorithm performance. Our contribution is based on realizing the automatic classification of concrete ultrasonic detection signals and improving the diagnostic accuracy of the detection results on previous machine learning methods.

The paper is organized as follows: Section 2 describes the algorithm involved in this paper in detail, especially the structures and steps in the algorithm; Section 3 describes the experimental objects, data, and algorithm parameter settings; Section 4 has our experimental results and the relevant analysis of algorithm performance; finally, the correlational experimental results and conclusion are given in Section 5 and Section 6.

## 2. Algorithm Descriptions and Basic Steps

The proposed method mainly includes two parts: signal processing and recognition. First, the WPT sub-algorithm filters out the high-frequency noise of detection signals and effective information is retained. Following this, the CNN sub-algorithm can automatically extract signal features and classify input signals. In the following subsections, two sub-algorithms are described in detail.

### 2.1. Wavelet Packet Transform Sub-Algorithm

The wavelet packet transform extracts useful information from signals with non-stationary characteristics and can provide arbitrary time-frequency resolution, which introduces a scale factor to adjust the time-frequency window, improves the resolution of frequency in the low-frequency range and time in the high-frequency range, and processes different frequency components effectively [25]. The idea of the WPT sub-algorithm can be understood as realizing the decomposition and reconstruction of ultrasonic detection signals through a set of filters [26].

**Figure 1:**
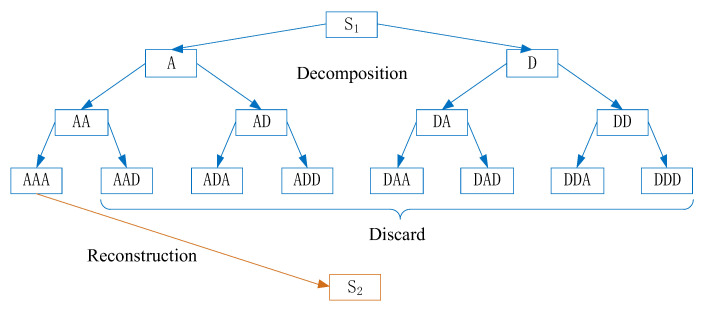
Decomposition and reconstruction of WPT sub-algorithm.

As a typical time-frequency analysis technique, wavelet transform has excellent localization properties in both frequency and time domains. It not only analyzes signals within a specified frequency band, but also analyzes signal components within a specified time period [11]. We adopt a working frequency of 50 kHz and a sampling frequency of 1 MHz in the ultrasonic detection system of this paper. Three-layer wavelet packet transform is used to decompose and reconstruct the signal. According to the wavelet packet transform steps, this completely locates the main frequency band of the received signal in the first node. The schematic diagram of the three-layer decomposition and signal reconstruction of the WPT sub-algorithm in this paper is illustrated in Figure 1. The blue mark in the diagram is the decomposition process, and the orange is the reconstruction process.

The decomposition and reconstruction steps of WPT sub-algorithm are as follows:

(1) The frequency band 0~*fs* of the ultrasonic detection signal S_1_ is divided into two equal parts by a pair of the low-pass filter and high-pass filter, a low frequency (A) band of 0~*fs*/2 and a high frequency (D) of *fs*/2~*fs*.
(2) Similarly, we perform 1/2 sampling of the decomposed low-frequency band and high-frequency band, respectively. Then a set of the low-pass filter and high-pass filter of which the frequency band (A) 0~*fs*/2 is divided into a frequency band (AA) of 0~*fs*/4 and a frequency band (AD) of *fs*/4~*fs*/2. Then, the frequency band (D) of *fs*/2~*fs* is divided into (DA) of *fs*/2~3 *fs*/4 and frequency band (DD) of 3 *fs*/4~*fs*.
(3) Repeat calculate decomposition as in step 2, then obtain each sub-signal of the ultrasonic detection signal with equal bandwidth and non-overlapping frequency bands.
(4) Reconstruct the complete signal S_2_ using the wavelet packet coefficients of the first node in the third layer.

In the process of decomposition, the time-frequency spectrum generated by choosing different wavelet packet basis functions and the number of decomposition layers is also different [27,28]. There is no universally recognized theory for the selection of wavelet basis functions based on present research results and engineering experience. It is well known from practical engineering applications that the choice of wavelet function usually varies with the signal type and processing purpose. Daubechies proposed the Daubechies (dbN) wavelet function, where N is the order of the wavelet. This wavelet function is widely used in the field of ultrasonic signal analysis and damage identification [27], and has comprehensive and excellent properties. Daubechies (db) wavelet function has good compact support, approximate symmetry, and smoothness [29]. Different orders of db wavelet decomposition have been applied to detection signals, and db15 is still selected by the trial-and-error method in WPT sub-algorithm to decompose and reconstruct the ultrasonic detection signals [12,24].

### 2.2. Convolutional Neural Network Sub-Algorithm

Mechanism of convolutional neural network (CNN) imitates the natural visual cognition of biology [30], and a deep learning network model generally is used for image convolution calculation. A typical CNN structure is mainly composed of convolutional layers, pooling layers, and fully connected layers [31]. The convolutional layers can obtain a set of optimal convolution kernels that meet the minimum loss function through training and use the convolution kernel to realize automatic feature extraction. The pooling layer can extract the most important features from the convolutional layer and perform dimensionality reduction operations in the time dimension. The stacking of the convolutional layer and the pooling layer forms a deep network structure, where high-level sequence features are extracted through layer-by-layer abstraction calculation. One-dimensional Convolutional Neural Network (1DCNN) is a form of CNN. The typical CNN is mainly used for feature recognition of two-dimensional images, while 1DCNN has only one dimension, so it is widely used for feature recognition and extraction of time series. Structurally speaking, 1DCNN also contains convolutional layers and pooling layers, and a fully connected layer, which outputs the results.

**Figure 2:**
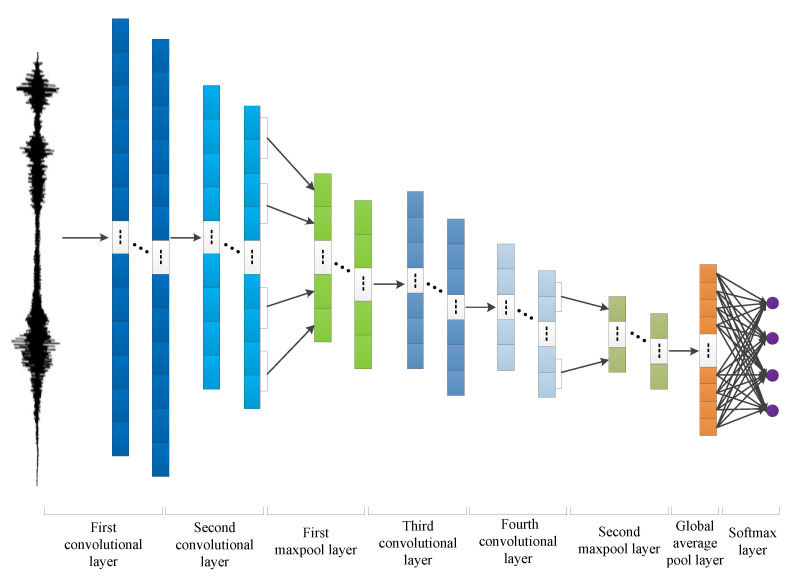
One-dimensional convolutional neural network structure diagram.

The specific structure of the CNN sub-algorithm is shown in Figure 2. It consists of four convolutional layers, two maximum pooling layers, one global average pooling layer, and one softmax layer. In the structure of CNN sub-algorithm, the global average pooling layer (GAP) is used to replace the fully connected layer [32]. The advantages of GAP are observed in that the conversion between the feature map and the final classification of GAP is easier, and GAP does not have a lot of training for tuning the parameters. To the reduced spatial parameters will make the model more effective in resisting over-fitting. Furthermore, in order to prevent the over-fitting phenomenon of deep neural networks, regularization methods can be used to enhance the generalization ability of the model. We adopt the dropout method in the softmax layer, where some neurons are temporarily discarded from the neural network according to a certain probability during neural network training. When the neural network is tested, the abandoned neurons are restored.

The main steps of CNN sub-algorithm are as follows.

(1) Initialize the network. Set the network structure and initial weights.
(2) Input data. The one-dimensional signal data are used as the input of the network to start the training of the network.
(3) Execute convolution and pooling operations. The input data are calculated and propagated forward through the convolutional layers, the maximum pooling layers, and the global average pooling layer. Then, the output layer generates a value.
(4) Calculate the network error. Calculate the error between the output value and the target value.
(5) Update weights. When the error is larger than the expected value, the error is transmitted back to the network, and the errors of the global average pooling layer, the maximum pooling layers, and the convolutional layers are sequentially obtained. Calculate new weights of the network according to the error and repeat step 3.
(6) Stop running. When the output error is less than the expected value or reaches the maximum epoch, the calculation of CNN sub-algorithm stops.

### 2.3. Stochastic Configuration Network

Stochastic configuration network (SCN) is a type of randomized learning model. SCN is a standard three-layer forward feedback network structure composed of an input layer, a hidden layer, and an output layer. The parameter value ranges of input weight, threshold, and hidden layer node number parameter value range depend on the training data and need to meet the constraints of the inequality supervision mechanism to ensure that the SCN has universal approximation capability for any given nonlinear mapping function [25]. The specific steps are as follows in [25].

(1) Suppose that a single layer feed-forward network is built with *L*-1 hidden nodes, calculate the network output error. If the error fails to satisfy the given error criterion, then a new hidden node must be added, i.e., a set of weights and biases is added.
(2) When adding a new hidden node, the input weight and bias vectors are randomly generated. The randomly generated vectors must satisfy the constraint of inequality.
(3) Calculate the output weights of the current network.
(4) Update the output weight and use the standard least squares method to compute the output weight.
(5) Calculate the error in step *L*. If it is less than the error criterion given in advance, then the SCN model training is completed. Otherwise, continue to add hidden layer nodes according to step 2 until the error criterion is met or the set maximum number of hidden layer nodes is reached. When a new hidden node is generated, the parameters of other nodes that have been determined remain unchanged.

### 2.4. Model Evaluation Indices

**Figure 3:**
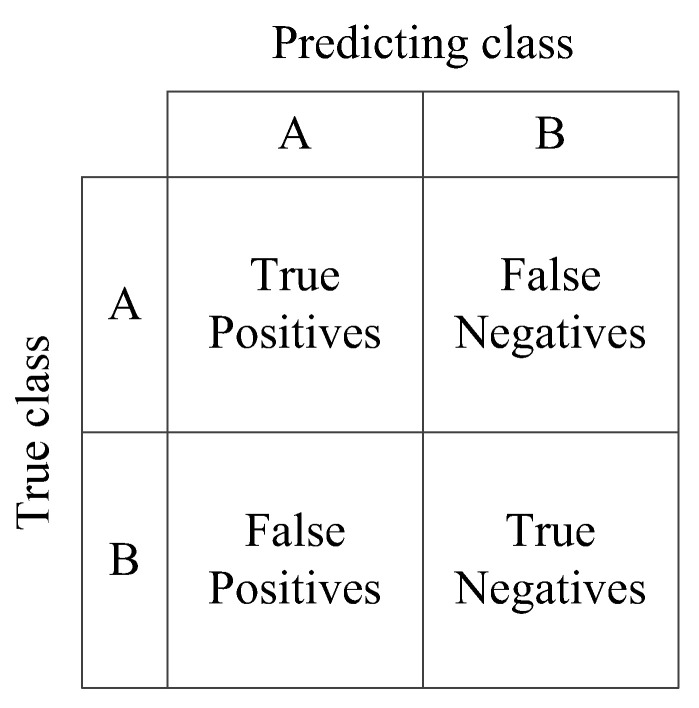
Schematic diagram of classification confusion matrix.

In addition to the commonly used accuracy (*Acc*) rates in model evaluation indices, precision (*P*) and recall (*R*) are used to help to analyze the test results [33]. The precision and recall can evaluate the recognition performance of the model on each category of the classification task. The precision indicates the ratio of the correct number to the total number of prediction results. The recall indicates the ratio of the correct number of predictions to the total number of true results. The two indicators are represented by the confusion matrix in Figure 3.

In Figure 3, A and B represent different categories. True positives (*TP*) represent true cases, i.e., positive samples are correctly identified as positive samples. False positives (*FP*) represent false positive cases, i.e., negative samples are incorrectly identified as positive samples. False negatives (*FN*) represent false negatives, i.e., positive samples are incorrectly identified as negative samples. True negatives (*TN*) represent true negatives, i.e., negative samples are correctly identified as negative samples.

The calculation equations of model evaluation indices are:

(1) $$Acc=\frac{TP+TN}{TP+FN+FP+TN}$$

(2) $$P=\frac{TP}{TP+FP}$$

(3) $$R=\frac{TP}{TP+FN}$$

To comprehensively evaluate the classification performance of the model based on the precision and the recall, an evaluation index F-score is introduced. The *F* metric value is the weighted harmonic average of the precision and the recall rate under the non-negative weight *β*. The higher the value, the better the classification performance [34]. The calculation equation is as follows:

(4) $$F_{\beta }=\frac{(1+\beta^{2})\times P\times R}{(\beta^{2}\times P)+R}$$

where *β* takes 1, which means that the precision is as important as the recall rate.

### 2.5. The Process and Steps of the Proposed Method

**Figure 4:**
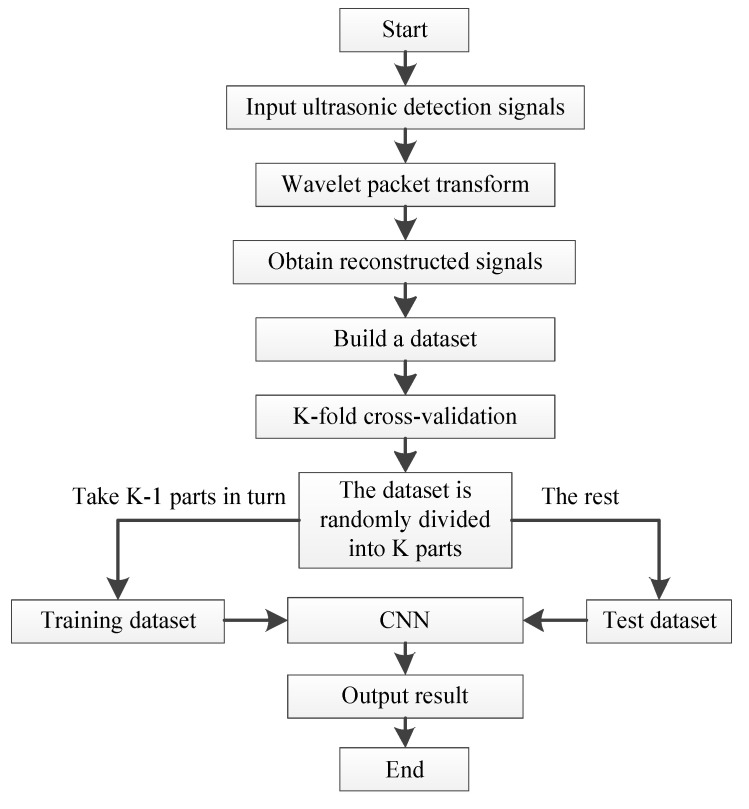
Flow chart of the proposed classification method.

The overall process of the concrete ultrasonic detection signal classification method described in this paper is shown in Figure 4.

The main steps of the proposed method are as follows.

(1) After the detection signal is decomposed by db15 wavelet packet in three layers, the signal components of eight different frequency bands are obtained, and the wavelet packet coefficients of the first node in the third layer are extracted to fully reconstruct the fundamental signal.
(2) Combine K-fold cross-validation method to divide the reconstructed dataset into the training dataset and the test dataset.
(3) Execute the CNN sub-algorithm to build the classification model. Input the data sets of step 2 to train, test and obtain the classification models.

## 3. Experimental Environment and Algorithm Parameter Settings

**Figure 5:**
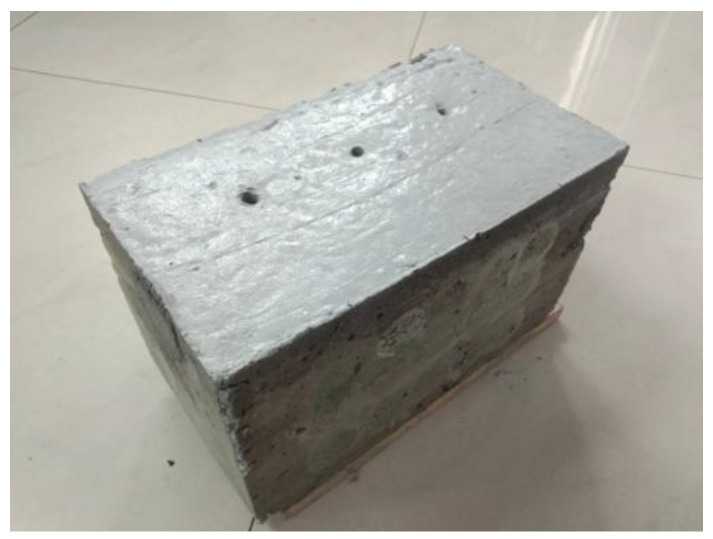
Concrete block I.

**Figure 6:**
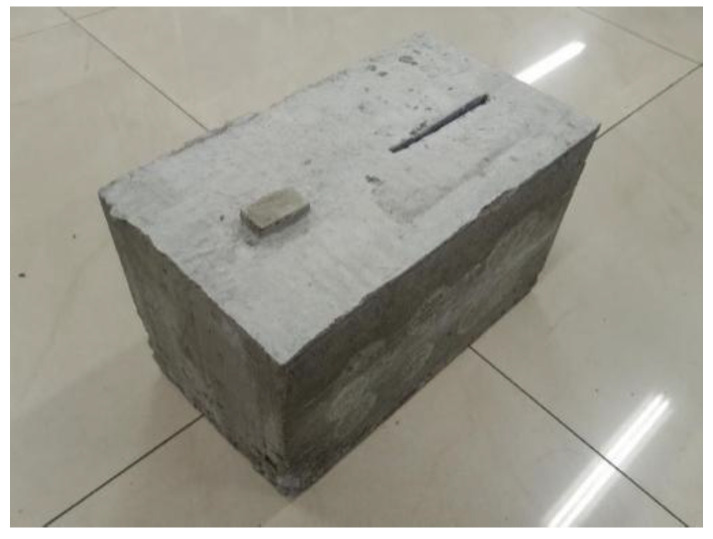
Concrete block II.

The test data are from the ultrasonic testing experiment, 50 kHz low-frequency ultrasonic probes are used, and the detection method is the ultrasonic transmission method. We applied couplant on the surface of detection positions of the concrete to remove the air between probes and the concrete blocks, so that the ultrasonic probe can penetrate the concrete effectively. The experiment objects used for ultrasonic testing in this case study are two C30 class concrete blocks shown in Figure 5 and Figure 6. All concrete test blocks are made by mixing ordinary Portland cement type I, water, sand, and gravel at the ratios of 461, 175, 512, and 1252 kg/m^3^, respectively. The sizes of the two concrete samples are the same, 300 × 200 × 200 mm. One of the concrete blocks contains three sizes of hole defects with diameters of 5 mm, 7 mm, and 9 mm. The distance between two penetrating holes is 85 mm, which is shown in Figure 5. Another concrete block contains crack and foreign body defects. The size of the crack is 100 × 3 mm. The size of the foreign matter of wood is 40 × 20 mm. The distance between the two defects is also 85 mm. The material object is shown in Figure 6. The defects penetrate the concrete blocks so that we can observe the actual locations of the defects.

**Figure 7:**
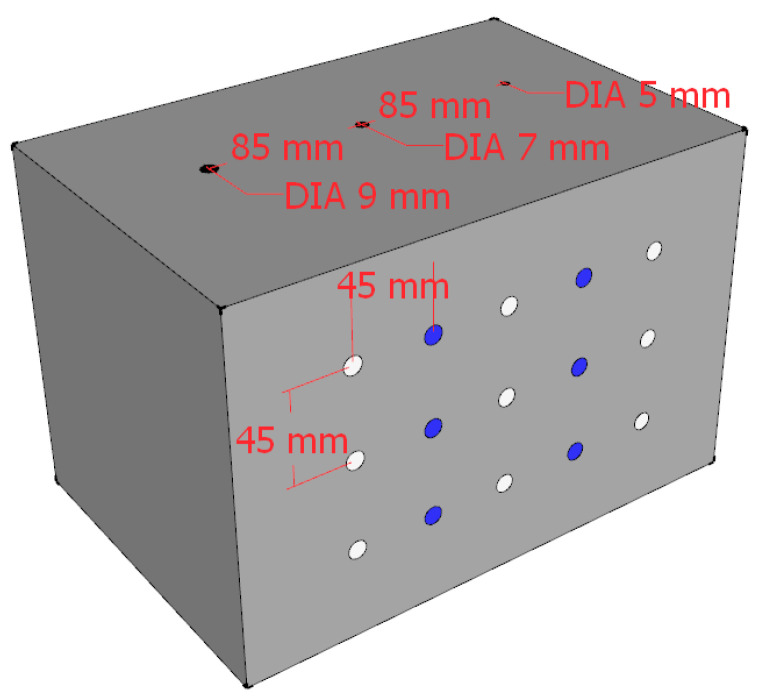
Block I detection position.

**Figure 8:**
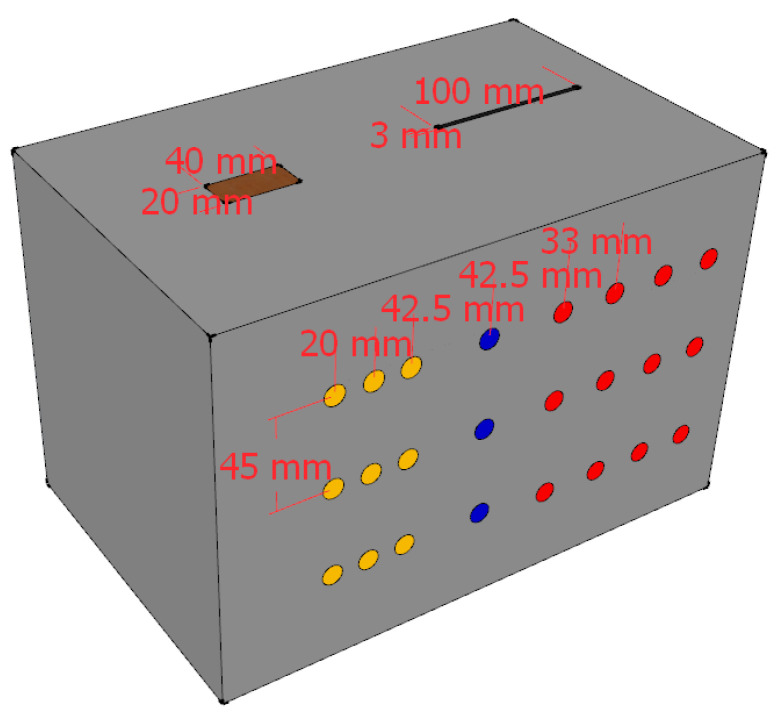
Block II detection position.

**Figure 9:**
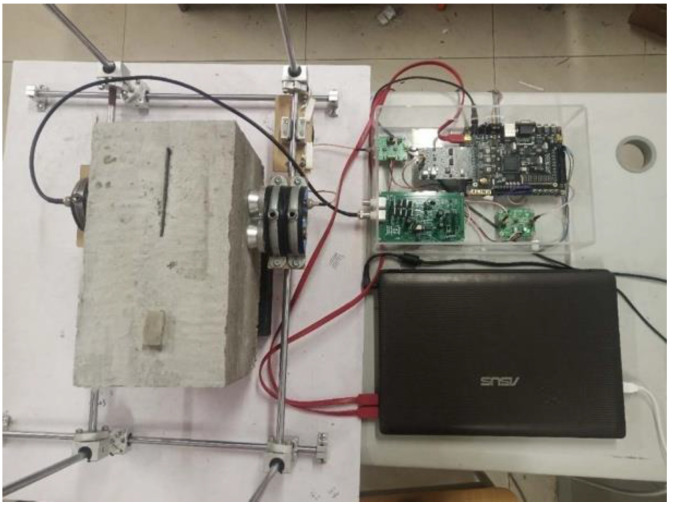
Ultrasonic detection hardware system.

Figure 7 and Figure 8 show the schematic diagrams of the detection positions. In the figures, the blue points, white points, red points, and yellow points locate the detection positions of no defect, hole, crack, and foreign matter, respectively. The center distances between the detection points arranged on the surface of the concrete block are shown in Figure 7 and Figure 8. Figure 9 shows the ultrasonic detection hardware system used in our experiments. The working performance of the hardware system that we evaluated in the published paper is reliable. The receiving end of the detection system is equipped with a spare probe. In the experiment, the transmitter produces ±100 V square wave pulse signal is used to excite the ultrasonic probe, and the signal sampling frequency at the receiver is 1 MHZ. The size and category of datasets are shown in Table 1. Each sequence contains three cycles of ultrasonic detection signals, with a total of 12,000 sampling points.

The proposed algorithm proposed in this article runs on a computer with Windows 10 64-bit operating system. The CPU specification is 6-core 2.08 GHz Inter Core i5-8400, and the memory specification is 32 GB 2400 MHz DDR4. The software used includes MATLAB 2014b and Python 3.7. The main parameters setting of the WPT-CNN algorithm are listed as follows.

The main parameters of the WPT sub-algorithm are: db15 is used as the wavelet basis function to perform 3-layer decomposition; Shannon entropy is calculated and used as the optimal wavelet basis. The fundamental frequency of ultrasound in this paper is 50 kHz. According to the frequency range of each node after decomposition, the first node of the third layer is extracted to obtain the reconstructed signal.

The parameters of K-fold cross-validation method are set as follows: K = 5, N = 5400. K represents the number of divided data and the number of classification experiments, and N represents the total amount of data. Under K-fold cross-validation, the data are randomly divided into five parts, four parts with a total of 4320 sequences are the training dataset, and the remaining part with a total of 1080 sequences are the test dataset. To adjust the hyperparameters of the model and to conduct a preliminary evaluation of the model’s ability, we select 620 sequences from the training set as the validation set. For the four-classifying model, data from three sizes of hole defects are trained and identified as the same kind. For the six-classifying model, data from three sizes of hole defects are trained and identified as three kinds of defects. For the five-classifying model, data from 5 mm hole defects are not used for the training dataset and validation set but for the test dataset, and they are expected to be identified as two kinds of hole defects.

The network structure of CNN sub-algorithm is shown in Figure 2 in Section 2. The parameters of the CNN sub-algorithm are as follows. We set the number of training times to 200 and the batch size to 100. The first layer of convolutional layer has 64 convolution kernels, the size is 8, the step size is 4, and the filling method is the same. The parameters of second convolutional layer are the same as the first layer. The first layer of pooling layer adopts the maximum pooling method, the size of the convolution kernel is 2, and the step size is 1. The third convolutional layer has 128 convolution kernels, the size of the convolution kernel is 4, and the step size is 2, and the filling method is the same. The fourth convolutional layer has the same parameters as the third, and the second pooling layer uses the maximum pooling method. The above layers all use the rectified linear unit (ReLU) as the activation function. The third layer of the pooling layer adopts the global average pooling method, and the node retention probability of random inactivation (dropout) is set to 0.3; the last softmax layer has four nodes, and the softmax function is used. When training the model, we use the cross entropy as the loss function, we use the Adam optimizer, and set the learning rate to 0.001.

The stochastic configuration network parameters are set as follows [12]. The maximum number of hidden layer nodes *L* is 200 (one epoch is performed when *L* is accumulated for one cycle), the training error is 0.01, and the activation function is the Log-sigmoid transfer function (logsig). The maximum number of random configuration is 100, the random weight range is {0.5, 1, 5, 10, 30, 50, 100}, the inequality constraint coefficient is {0.9, 0.99, 0.999, 0.9999, 0.99999, 0.999999}, set step length to 1 as the number of hidden layer nodes is accumulated.

## 4. Experimental Results and Analysis

### 4.1. Reconstructed Signals by the WPT Sub-Algorithm

**Figure 10:**
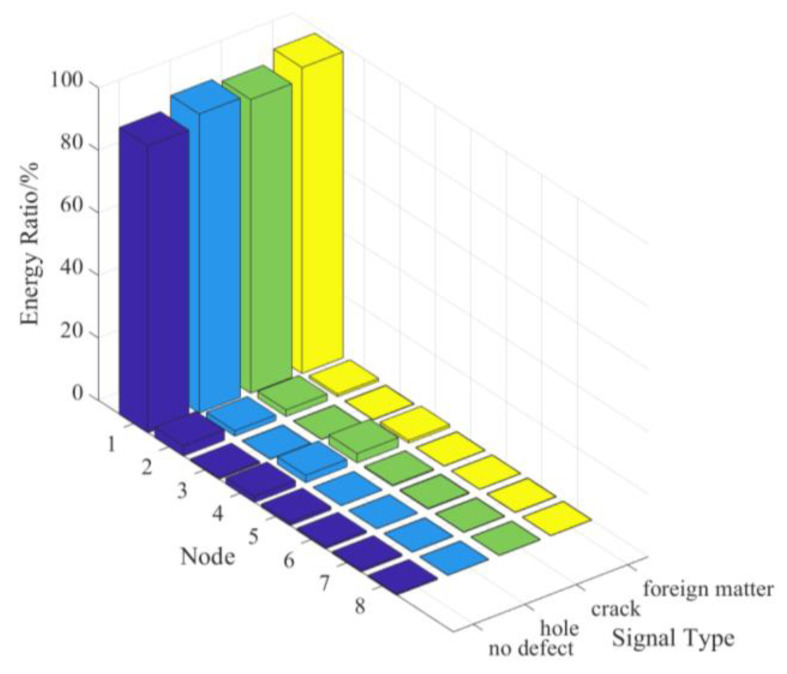
The single cycle ultrasonic signal.

After the three-layer decomposition of the wavelet packet is processed, the energy ratio of the signal component in each node is calculated, which is the sum of the squares of the wavelet packet coefficients, as shown in Figure 10. The greater the energy, the higher the value of the wavelet packet coefficients [35]. Based on the characteristic of energy distribution, the fundamental frequency node of the signal is self-explanatory. Naturally, the first node coefficients of the third layer are used to reconstruct them into complete signals due to this result.

**Figure 11:**
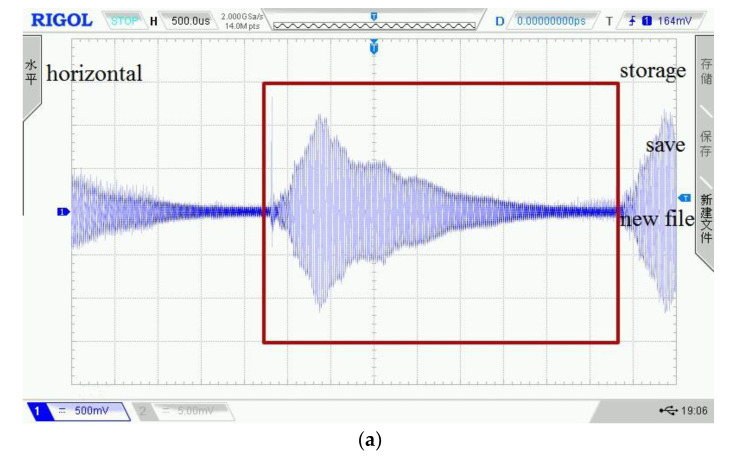

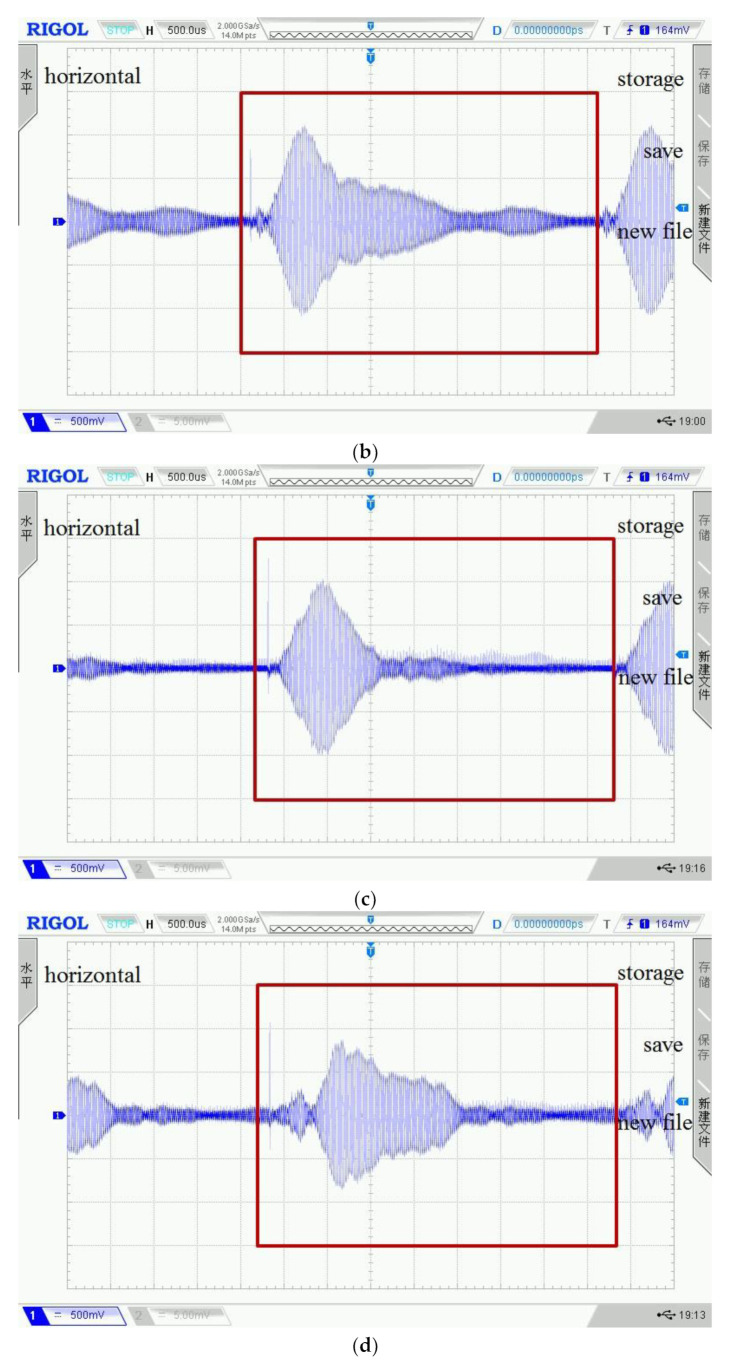
(**a**) No defect detection waveform; (**b**) Hole detection waveform; (**c**) Crack detection waveform; (**d**) Foreign matter detection waveform.

**Figure 12:**
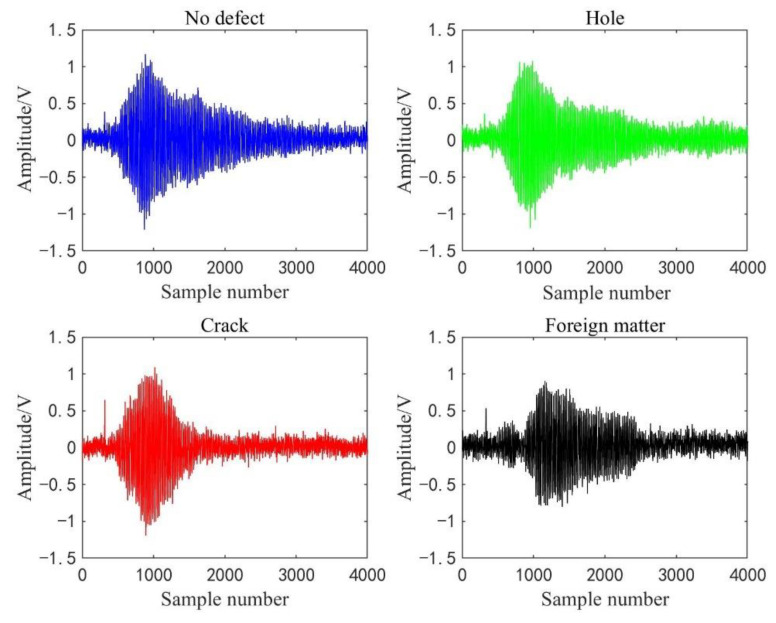
Four raw waveforms of detection signals.

**Figure 13:**
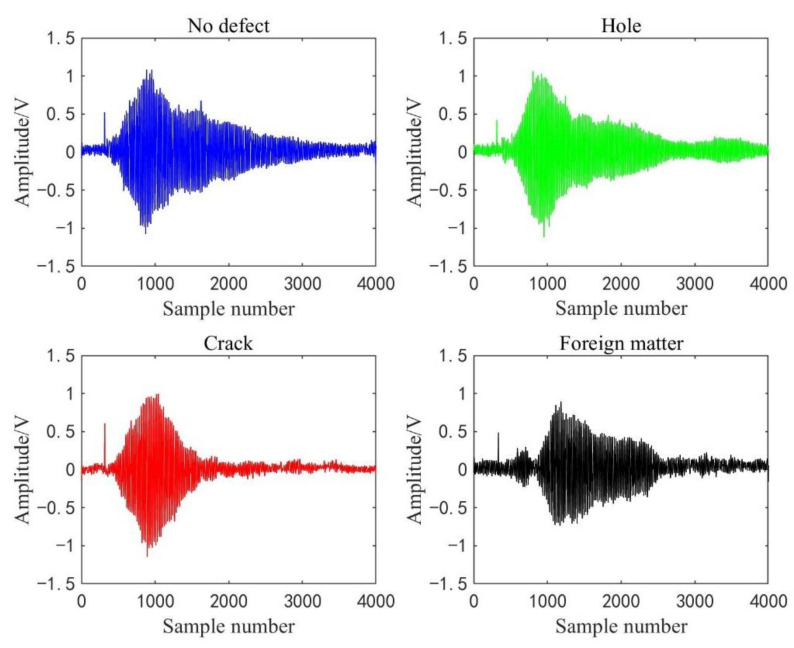
Four reconstruction waveforms of detection signals.

Figure 11 shows the waveforms of four kinds of detection signals displayed on the oscilloscope, where the wave in the red box is a complete cycle signal data sequence. In addition, four displayed waveforms are randomly selected from the detection dataset of four kinds of concrete conditions. To display the waveform curves before and after processed by the WPT sub-algorithm, we draw the last cycle of the selected signals in Figure 12 and their reconstructed waveforms in Figure 13. It shows that the reconstructed detection signal waveforms are smoother by comparison with Figure 12 and Figure 13. High-frequency noise is almost removed from the reconstructed waveform, which perhaps reduces the impact of high-frequency noise on the accuracy of the classification model.

### 4.2. Four-Classifying Model

The five-fold cross-validation method randomly divides the sample set into a training dataset (including validation dataset) and test dataset [36]. Each subset is taken out of the total sample set by uniform random sampling to ensure that the proportion of sub-samples is consistent with the overall sample. As is well known, the training dataset is used to optimize model parameters, the verification set is used to adjust hyperparameters, and the test dataset is used to verify the generalization ability of the model [37,38]. When the CNN sub-algorithm is applied to concrete ultrasonic detection signals to build the four-classifying model, the stochastic configuration network algorithm is introduced as a comparison model simultaneously. To analyze the performance of the CNN model, the error and accuracy change curves were illustrated during the training process in an experiment with the highest test accuracy.

**Figure 14:**
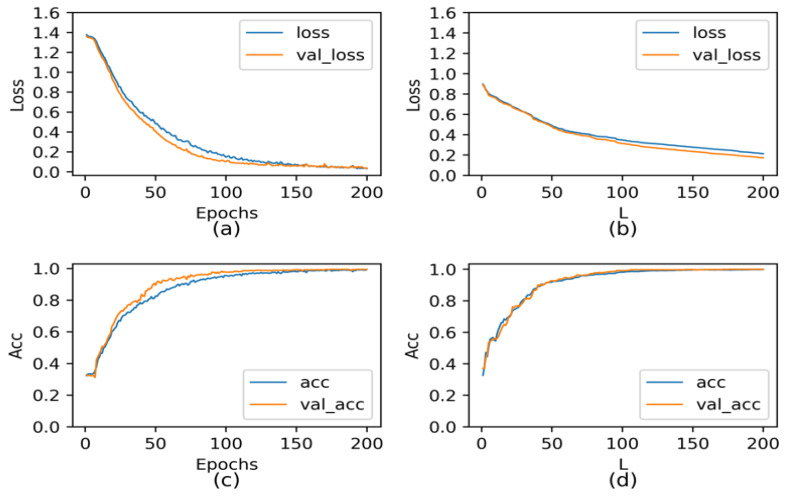
(**a**) Error curves of CNN; (**b**) Error curves of SCN; (**c**) Accuracy curves of CNN; (**d**) Accuracy curves of SCN.

As shown in Figure 14a,b, the error curves of two models on the training dataset and validation dataset are plotted, respectively, where the blue curve is the error change curve of the training dataset and the orange curve is the error change curve of the validation dataset. Figure 14c,d shows the classification accuracy change curves of CNN and SCN models on the training set and validation set. It can be seen from Figure 14 that the rate of convergence of CNN model is less than that of SCN model, and the final training error is less than that of SCN model. The classification accuracies of CNN on the training dataset and validation dataset during the training process are both higher and close to 100%. With the increase of epoch, the classification accuracy of SCN model on the training set tends to be stable.

The highest, lowest, and average accuracy rates among the five results of the two models are listed in Table 2, owing to five-fold cross-validation. The recognition accuracy values of five CNN models all exceeded 99%. The difference between the highest accuracy and the lowest accuracy is only 0.28%, which is very small and reflects the imitative effect. The comparison with the recognition accuracy of SCN shows they are lower than the CNN model, and their difference is around 1%. Then, CNN model has higher classification accuracy for concrete ultrasonic detection signals and is more stable, but it needs a lot more time for training and testing. In this paper, the processor of computing environment is CPU. High-performance computing equipment such as GPU, has been used for intelligent computing, and the running time of CNN model will be greatly reduced. Similarly, if the time cost is acceptable, then the application of CNN sub-algorithm to ultrasonic testing equipment will not be so difficult.

Table 3 shows the accuracy, recall, and F-score of two models are calculated for evaluating performance on different detection signal types. The four indices of CNN model are higher than those of SCN model, especially for the recognition accuracy of detection signals of the foreign matter defect. CNN models have the highest overall classification accuracy on four types of detection signals.

The recognition accuracy of CNN model of the concrete ultrasonic detection signal is higher than 99%, but still cannot reach 100%. Furthermore, it is true that when detecting similar defects in different positions of concrete blocks, the obtained detection signals have uncertain differences. For example, the built dataset contains 200 data samples detected from four edges of crack and foreign matter defects. These signals are quite different from signals detected from the middle positions. Spontaneously, this leads the recognition accuracy of the classification model not to reach 100%. On the other hand, there are some random links in the operation of neural networks, such as the initial weights and thresholds of CNN and SCN, the randomness of regularization, etc. [39,40], which will cause the model classification accuracy fluctuate every time. From another perspective, the classification results processed by five-fold cross-validation, fluctuate in a small range, and indicate that the CNN model has strong stability and generalization ability.

### 4.3. Six-Classifying Model

The six-classifying experiment for CNN model is analogous to the above four-classifying experiment, but three sizes of hole defects are treated as three different defects. In this classification experiment, the five-fold cross-validation partition datasets for training and testing are still used. Finally, five test results from the CNN and SCN models are obtained. The highest, lowest, and average accuracy rates among the five results of the two models are listed in Table 4, and the precision, recall, and F-score of the two models are listed in Table 5.

The running times of ten models are essentially unchanged because the data size has not increased. The lowest accuracies of the two models both reach more than 95%. In addition, CNN classification performance is better than in the SCN models according to four evidence indices. Compared with the four-classifying experiment, the recognition accuracies of this experiment decreased slightly. One reason is that the data size of three types of hole defect is about half lower than those of other three types of defects. In this experiment, the hole data are divided into three categories, so the complication of classification features of hole defeats is increased compared to that of the four-classifying CNN models and SCN models. Subjectively, it shows that the small difference of defect sizes makes their classification calculated by CNN models or SCN models more difficult. The results show that the proposed WPT-CNN method has a certain effectiveness in more complex detection and has higher practical engineering significance.

Some evaluation index values of the three sizes of hole defect decrease significantly compared with the values in Table 3; in particular, the recall of 5 mm hole defect is the worst. The reason for this is that CNN models confuse a 5 mm hole defect with the other five types of defects, and the 7 mm hole defect, 9 mm hole defect, and crack defect account for the largest number according to their precision and F-score values. Therefore, defect identification of different sizes will be the subject of one of our future studies.

### 4.4. Five-Classifying Model

Being curious about the recognition effect of the unknown detection data on WPT-CNN models, we proceed the five-classifying experiment. According to the five-classifying experiment, some interesting results and analysis are shown in this section.

The five-classifying experiment for CNN and SCN models is quite different from the six-classification experiment in that the dataset used for building models does not consist of 5 mm hole defect data. First, five types of data are used to build training, validation, and test dataset, where their data sizes are 3360, 480, and 960, respectively. The same data are from 1500 sequences from non-defective detection points, 1200 sequences from hole defect detection points (600 from 7 mm and 9 mm hole defects equally), 1100 sequences of crack defect detection data, and 1000 sequences of foreign matter defect detection data. Then, to train and test the five-classifying CNN and SCN models in the same way of four-classifying experiment. Then, all the 5 mm hole defect data are used as additional test dataset to obtain their identification results. Here, it is regarded as a positive result that the 5 mm hole defect data are recognized as a type of 7 mm or 9 mm hole defect data.

The recognition accuracies and evaluation indices of the five-classification experiment are listed in Table 6 and Table 7, respectively. Experimental results of testing 5 mm hole defect data are presented in Table 8, where the highest and lowest percentages are the highest and lowest positive percentages of 5 mm hole defect data that are correctly identified as two types of hole defects of five models. The average percent is the average value of each value across five experiments.

The data size of the dataset in this experiment is less than those of the above two experiments, so it takes less runtime to build the models. Compared with the recognition accuracies of six-classifying CNN and SCN models in Table 3, those in Table 6 are improved by about 1% on average. Then, the complexity of recognition of 5 mm hole defect data is evident. According to Table 6, it is obvious that CNN models have a higher recognition accuracy than SCN models, especially on foreign matter data. A reason is that part of the foreign matter dataset is collected from the foreign matter edges, which contain more uncertain noise and the time uncertainty of the waveform due to heterogeneous concrete structures. It is also a major challenge in the ultrasonic detection task.

The experiment results in six tables demonstrate the validity of WPT-CNN models and SCN models for labeled detection data types. However, it is normal that data are of unknown types, obtained in actual engineering detection tasks, such as defects of unknown size. The used models are established by the above five types of data to classify the 5 mm hole defect data. Then, the positive percentage of 5 mm hole defect is the sum of two positive percentages.

It has been shown that 5 mm hole defect data are more difficult to identify from the above six-classifying and four-classifying experiments. Similarly, the highest percentages for CNN model and SCN model to identify them as hole defects are only 41% and 36.83%, and their lowest percentages are 27.17% and 24.50%. Furthermore, both 5 mm hole defect and crack defect are small cavity defects, and perhaps have some common features owing to the time uncertainty of the waveform or noise. This is an important factor for CNN models classifying 5 mm hole defect data as crack defect data at approximately 40%. Nevertheless, the CNN models identify them as the other three types of defects more than 70% of the time, which proves that the WPT-CNN algorithm has a certain effectivity to identify defect signals of unknown types. It is worthwhile to deeply study the intelligent recognition of unknown defects data.

## 5. Correlational Research and Experiment

### 5.1. Raw Dataset Recognition Experiment

When building CNN models, the original signals can be directly used as input data, which simplifies the process of pattern recognition [39]. However, the experimental data are from concrete ultrasonic detection signals, which have uncertain noise and the time uncertainty of the waveform due to concrete structures. To analyze the influence of denoizing on the recognition performance of the WPT-CNN model, the raw data were used for four-classifying CNN model training and testing. Similarly, SCN and CNN models are built to perform classification experiments with the five-fold cross-validation method.

In the experimental results, the highest recognition accuracy of CNN models is 98.5%, and the average recognition accuracy is 97.8%. In addition, the recognition accuracy of SCN model is lower than 93%. Compared with the signal recognition result of four-classifying WPT-CNN and WPT-SCN models, their accuracies reduce similarly, especially the average accuracy of SCN is reduced by 6.9%. The experimental results reveal that the noise in concrete detection signals will affect the recognition accuracy of WPT-CNN model. The recognition accuracy is improved by filtering out signal noise and invalid information before the classification task.

### 5.2. Recognition Experiment Based on Features Extraction

Extracting features is the critical step for traditional machine learning to achieve classification tasks. CNN sub-algorithm automatically extracts features through the mechanism of convolution operations, and it is not necessary to build a feature dataset for modeling. Because of this mechanism, a CNN model takes much more time than that of traditional machine learning. In this experiment, we try to extract effective features from signals for CNN modeling and improve its running speed after the WPT sub-algorithm processes. In this experiment, the SCN model is still used as the comparison model.

First, five features of ultrasonic detection signals and constructed feature datasets were extracted which are similar to the described operations in the paper of Zhao [11]. The CNN and SCN models trained by the feature dataset can only obtain the recognition accuracies of less than 80% for the recognition of four types of detection signals. Then, we increased the number of feature categories to 17 and constructed another feature dataset to perform the experiment again. The recognition accuracies of CNN and SCN trained by the 17-feature dataset are both around 97%, which is inferior to WPT-CNN four-classifying models. However, the CNN training time is reduced by 90% compared with the time listed in Table 1 and the SCN training time is only reduced by 5%.

On all these counts, the feature extraction methods of the CNN sub-algorithm need to be studied more intensively. The results of relative recognition experiments show that the proposed WPT-CNN algorithm is the best method with the highest accuracy.

## 6. Conclusions and Future Works

The proposed method to automatically classify and recognize concrete ultrasonic detection signals comprises the wavelet packet transform to decompose the original detection signal into three layers and obtain the wavelet packet coefficient of the first node in the third layer to reconstruct signals, and a one-dimensional convolutional neural network to classify and recognize the reconstructed signal data. Four identification experiments on 5400 detection data samples and a four-classifying experiment were primarily carried out, which constitutes four datasets with no defects, holes (three diameter holes), cracks, and foreign matter. Through experiments on the datasets constructed with four processing methods, WPT-CNN has the highest recognition accuracy and best generalization ability estimated by the evaluation indices, and comparison results of the stochastic configuration network. The precision and recall of the four types of signals exceed 99%, and the F-score is more than 0.99. The WPT-CNN method can effectively identify the defect signal and deploying it in the ultrasonic testing instrument can solve the current shortcomings. On the other hand, the experiment results of six- and five-classifying models, and related research on raw data and feature extraction recognition revealed the cause-and-effect factors affecting recognition performance.

Nevertheless, there are still four major challenges in applying the algorithm in this paper to ultrasonic testing instruments: data size, data nature, modeling challenges, and uncertainty [15]. Obtaining a larger amount of data based on a variety of reliable ultrasonic detectors will be a necessary basis for algorithm application research. In the follow-up research, we will continue to optimize the algorithm model and study the application of other deep learning models, which is also an important task. The proposed method was proven to be effective for the ultrasonic detection signal recognition of C30 class concrete, and the research objects should be extended to more classes of concrete. Debonding between rebars and concrete, a more complex defect, is also an important research topic for the future [41]. In addition, the application of the proposed method to actual detection instruments is the ultimate goal of the future.

## Figures and Tables

**Table 1:** The size and category of datasets.

Category	No Defect	Hole			Crack	Foreign Matter	Total
		5 mm	7 mm	9 mm			
Size of dataset	1500	600	600	600	1100	1000	5400

**Table 2:** Recognition accuracy and average running time of four-classifying models.

Model	Highest Accuracy	Minimum Accuracy	Average Accuracy	Training Time/s	Test Time/s
CNN	99.91%	99.63%	99.78%	3042.5314	6.4150
SCN	98.98%	97.78%	98.53%	73.1314	0.0361

**Table 3:** Average values of four-classifying model evaluation indices.

Category	Model	No Defect	Hole	Crack	Foreign Matter
Precision	CNN	99.86%	99.77%	99.63%	99.80%
	SCN	99.13%	98.57%	98.10%	98.08%
Recall	CNN	99.53%	99.94%	99.81%	99.80%
	SCN	99.33%	99.61%	98.09%	95.90%
F-score	CNN	0.9969	0.9986	0.9972	0.9979
	SCN	0.9923	0.9908	0.9809	0.9696

**Table 4:** Recognition accuracy and average running time of six-classifying models.

Model	Highest Accuracy	Minimum Accuracy	Average Accuracy	Training Time/s	Test Time/s
CNN	99.16%	97.50%	98.33%	3064.5374	6.2243
SCN	97.68%	95.27%	96.90%	78.9466	0.0632

**Table 5:** Average values of six-classifying model evaluation indices.

Category	Model	No Defect	Hole			Crack	Foreign Matter
			5 mm	7 mm	9 mm		
Precision	CNN	99.67%	99.82%	95.26%	94.78%	98.99%	99.02%
	SCN	97.89%	96.84%	94.45%	93.51%	97.97%	98.07%
Recall	CNN	99.80%	89.33%	100%	99.50%	98.64%	99.50%
	SCN	99.35%	90.38%	98.68%	98.52%	95.78%	96.40%
F-score	CNN	0.9973	0.9425	0.9757	0.9708	0.9881	0.9926
	SCN	0.9860	0.9344	0.9649	0.9594	0.9685	0.9722

**Table 6:** Recognition accuracy and average running time of five-classifying models.

Model	Highest Accuracy	Minimum Accuracy	Average Accuracy	Training Time/s	Test Time/s
CNN	99.58%	99.17%	99.35%	2389.7757	5.4701
SCN	98.64%	97.29%	97.89%	65.4367	0.0411

**Table 7:** Average values of five-classifying model evaluation indices.

Category	Model	No Defect	Hole		Crack	Foreign Matter
			7 mm	9 mm		
Precision	CNN	99.60%	99.01%	99.83%	99.27%	99.00%
	SCN	98.89%	98.20%	99.02%	97.90%	95.62%
Recall	CNN	99.60%	99.83%	99.17%	99.00%	99.20%
	SCN	98.58%	100%	99.50%	97.32%	95.20%
F-score	CNN	0.9960	0.9942	0.9950	0.9914	0.9910
	SCN	0.9873	0.9909	0.9926	0.9758	0.9532

**Table 8:** Recognition results of the 5 mm hole defect data.

Model	Percent	No Defect	Hole		Crack	Foreign Matter
			7 mm	9 mm		
CNN	Highest	25.33%	29.33%	11.67%	33%	0.67%
	Minimum	30.17%	21%	6.17%	40%	2.67%
	Average	21.11%	25.43%	9.60%	43.17%	1.00%
SCN	Highest	34.17%	31.83%	5%	6.17%	22.83%
	Minimum	24.83%	13.83%	10.67%	37.33%	13.33%
	Average	26.73%	23.43%	6.13%	23.57%	20.13%
